# Unveiling the Polypharmacological Potency of FDA-Approved Rebamipide for Alzheimer’s Disease

**DOI:** 10.3390/ph18060772

**Published:** 2025-05-22

**Authors:** Israa J. Hakeem, Hadil Alahdal, Hanadi M. Baeissa, Tahani Bakhsh, Misbahuddin Rafeeq, Alaa Hamed Habib, Mohammed Matoog Karami, Maryam A. AL-Ghamdi, Ghadeer Abdullah, Abeer Al Tuwaijri

**Affiliations:** 1 Department of Biological Sciences, College of Science, University of Jeddah, Jeddah 23218, Saudi Arabia; ijhakeem@uj.edu.sa (I.J.H.); hmbaeissa@uj.edu.sa (H.M.B.); tabakhsh@uj.edu.sa (T.B.); 2 Department of Biology, College of Science, Princess Nourah bint Abdulrahman University, Riyadh 11564, Saudi Arabia; hmalahdal@pnu.edu.sa; 3 Faculty of Medicine, King Abdulaziz University, Jeddah 21589, Saudi Arabia; marafeeq@kau.edu.sa (M.R.); ahabib@kau.edu.sa (A.H.H.); 4 Department of Clinical Physiology, Faculty of Medicine, King Abdulaziz University, Jeddah 21589, Saudi Arabia; mkarami@kau.edu.sa; 5 Department of Biochemistry, Faculty of Science, King Abdulaziz University, Jeddah 21589, Saudi Arabia; maaalghamdi3@kau.edu.sa; 6 Experimental Biochemistry Unit, King Fahd Medical Research Center, King Abdulaziz University, Jeddah 21589, Saudi Arabia; 7 Department of Biology, Howard University, Washington, DC 20059, USA; ksa.ghadeer@gmail.com; 8 Medical Genomics Research Department, King Abdullah International Medical Research Center, Ministry of National Guard Health Affairs (MNGH), Riyadh 11426, Saudi Arabia; 9 Department of Clinical Laboratory Sciences, College of Applied Medical Sciences, King Saud Bin Abdulaziz University for Health Sciences, Riyadh 11481, Saudi Arabia

**Keywords:** Alzheimer’s disease, multitargeted docking, FDA, Rebamipide, MD simulations

## Abstract

**Background:** Alzheimer’s disease (AD) is a multifactorial neurodegenerative disorder characterised by the accumulation of neurotoxic substances in the brain, ultimately leading to progressive cognitive decline. The complex aetiology and involvement of multiple molecular targets in AD pathogenesis have made discovering effective therapeutic agents particularly challenging. Targeting multiple proteins simultaneously with a single therapeutic agent may offer a promising strategy to address the disease’s multifaceted nature. **Methods:** This study employed advanced computational methodologies to perform multitargeted molecular docking of FDA-approved drugs against four key AD-associated proteins implicated in disease progression. Among the screened compounds, Rebamipide—a drug conventionally used for treating gastrointestinal disorders—demonstrated notable binding affinities across all targets. Pharmacokinetic predictions, interaction fingerprinting, WaterMap analysis, density functional theory (DFT) calculations, and 100 ns MD simulations were performed for each protein–ligand complex to evaluate its multitarget potential. **Results:** Rebamipide bound effectively to the NR1 ligand-binding core, suggesting modulation of glutamatergic signalling while reducing β-secretase production and regulating neurotransmitter homeostasis through inhibiting monoamine oxidase-A. Furthermore, Rebamipide enhanced cholinergic neurotransmission by inhibiting human acetylcholinesterase, potentially improving cognitive function. Pharmacokinetic analyses confirmed favourable drug-like properties. Molecular interaction fingerprints revealed consistent hydrogen bonding, hydrophobic contacts, and π-π stacking interactions. WaterMap analysis indicated thermodynamically favourable water displacement upon binding, enhancing ligand affinity. DFT analysis of Rebamipide showed a 4.24 eV HOMO-LUMO gap, with ESP values ranging from −6.63 × 10^−2^ to +6.63 × 10^−2^ A.U., indicating reactive sites. TDDFT predicted strong UV absorption at 314 nm with a peak intensity of ~6500 L mol^−1^ cm^−1^. MD simulations over 100 ns demonstrated minimal structural deviations and stable ligand–protein complexes, reinforcing its multitarget efficacy. **Conclusions:** The comprehensive in silico investigation highlights Rebamipide as a promising multitargeted therapeutic candidate for Alzheimer’s disease. Its ability to modulate multiple pathogenic pathways simultaneously underscores its potential utility; however, these computational findings warrant further experimental validation to confirm its efficacy and therapeutic relevance in AD.

## 1. Introduction

Alzheimer’s disease (AD) is a progressive and irreversible neurodegenerative disorder affecting millions worldwide, posing a significant global health challenge. It is the most common cause of dementia, accounting for approximately 60–70% of all dementia cases [1,2]. AD is characterised by the gradual decline in cognitive functions, including memory loss, impaired thinking, and behavioural changes, ultimately leading to the loss of independence and a significant burden on patients, caregivers, and healthcare systems [3]. The hallmark neuropathological features of AD include the accumulation of extracellular amyloid-beta (Aβ) plaques and intracellular neurofibrillary tangles composed of hyperphosphorylated tau protein in the brain. These pathological changes disrupt neuronal communication and connectivity, leading to synaptic dysfunction and cell death. Despite extensive research efforts and considerable advances in understanding the disease mechanisms, effective treatments for AD remain elusive [4]. The multifactorial nature of AD presents a complex challenge in developing therapeutics. The disease arises from genetic, environmental, and lifestyle factors, making it challenging to effectively target a single molecular pathway [5]. Therefore, there is a growing interest in exploring multitargeted therapies that can simultaneously address multiple pathological aspects of AD [6,7,8,9,10,11,12,13]. In recent years, computational approaches have emerged as valuable tools in drug discovery and development, allowing researchers to explore vast chemical spaces and screen potential compounds in silico before experimental validation [14,15]. One promising strategy is multitargeted docking, where molecular docking simulations are performed against multiple target proteins to identify compounds that can interact with them simultaneously [13,16,17,18,19,20,21]. This approach provides insights into potential drug candidates with multitargeted activities, offering a promising avenue for tackling complex diseases like AD. We have carefully selected four target proteins based on their known roles in AD pathogenesis and their potential as therapeutic targets [22].

The four proteins targeted in this study, namely the NR1 ligand-binding core, Human Beta Secretase (BACE1), Human Monoamine Oxidase-A (MAO-A), and Recombinant Human Acetylcholinesterase (AChE), play crucial roles in the complex and interconnected pathogenesis of AD. Understanding their roles and interactions is essential to comprehend the disease’s underlying mechanisms and potential multitargeted therapeutic interventions. NR1 ligand-binding core (PDBID: 1PBQ) is part of the N-Methyl-D-Aspartate (NMDA) receptor, which plays a crucial role in learning and memory [23]. Dysregulation of NMDA receptors and excitotoxicity are implicated in AD progression. Targeting the NR1 ligand-binding core may help inhibit Aβ aggregation and mitigate excitotoxicity-induced neuronal damage. The NMDA receptor function in AD is dysregulated, leading to abnormal synaptic signalling and calcium influx [24]. This imbalance contributes to excitotoxicity, a process where excessive calcium entry causes neuronal damage and cell death. The excessive accumulation of amyloid-beta (Aβ) peptides, characteristic of AD, further exacerbates excitotoxicity by promoting NMDA receptor overactivation. Human Beta Secretase (BACE1) (PDB ID: 4D89) is responsible for the initial cleavage of amyloid precursor protein (APP), generating soluble APP beta (sAPPβ) and the membrane-bound C-terminal fragment (C99) [25]. C99 is subsequently cleaved by gamma-secretase to produce Aβ peptides, including Aβ40 and Aβ42, which are implicated in AD pathology as they have a greater propensity to aggregate and form neurotoxic oligomers, leading to Aβ plaque formation [26]. The aggregation of Aβ peptides and the deposition of amyloid plaques in the brain are central events in AD pathogenesis, causing neuroinflammation and synaptic dysfunction. Human Monoamine Oxidase A (MAO-A) (PDBID: 2Z5X) is an enzyme involved in the metabolism of neurotransmitters, and its inhibition may contribute to the protection of neurons against Aβ-induced neurotoxicity [27]. In AD, the breakdown of neurotransmitters is disrupted due to increased MAO-A activity, leading to imbalances in neurotransmitter levels [28,29,30,31,32]. This imbalance contributes to neurotoxicity, oxidative stress, and inflammation, further exacerbating AD pathology. Human Acetylcholinesterase (AChE) (PDB ID: 4EY6) is an enzyme responsible for acetylcholine hydrolysis in the brain, and its inhibition can enhance cholinergic neurotransmission [33]. Cholinergic dysfunction is associated with memory impairment in AD. Modulating AChE activity may improve cognitive function in AD patients. Reduced acetylcholine levels contribute to cholinergic dysfunction, a hallmark of AD, leading to impaired cholinergic neurotransmission and cognitive decline, and for these cognitive deficits, AChE inhibitors are used to increase acetylcholine levels and improve cholinergic neurotransmission temporarily [26,34]. The four proteins, NR1 ligand-binding core, BACE1, MAO-A, and AChE, are interconnected in AD pathogenesis through intricate molecular pathways. The dysregulation of one protein can lead to alterations in other proteins and pathways, amplifying the disease process [35]. For instance, Aβ peptides, produced by BACE1 cleavage of APP, can induce excitotoxicity by overactivating the NMDA receptor via the NR1 ligand-binding core [36]. This excitotoxicity, in turn, contributes to the increased activity of MAO-A, leading to neurotransmitter imbalances and neurotoxicity. Furthermore, Aβ peptides can interact with AChE and enhance its activity, exacerbating the reduction in acetylcholine levels and aggravating cholinergic dysfunction in AD. Targeting multiple proteins simultaneously, as demonstrated in multitargeted docking studies, can profoundly impact AD pathogenesis more than single-target therapies [23,25,27,33].

**Figure 1:**
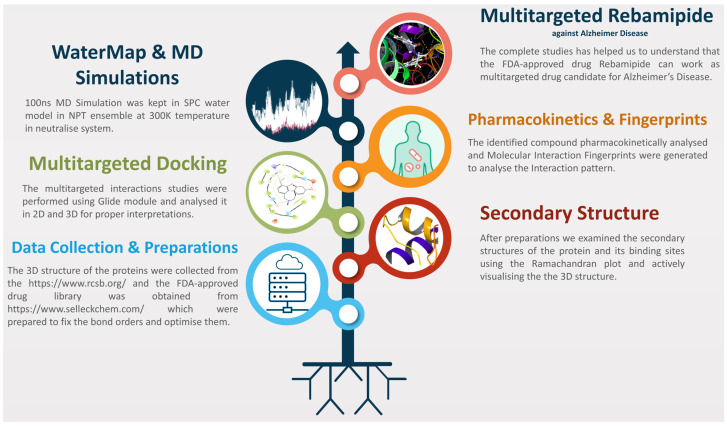
The workflow of the complete study shows the methods that were followed.

In this study, we aimed to employ multitargeted docking and molecular dynamics (MD) simulations to screen a diverse set of FDA-approved drugs against four critical AD proteins. After that, the identified compound was taken for pharmacokinetic studies, and the protein–ligand complex was used for pattern identification using the molecular interaction fingerprints. Further, we also performed the MD simulation for 100 ns for each complex to understand and analyse the deviation, fluctuations, and other possible interactions, as shown in the workflow in Figure 1.

## 2. Results

### 2.1. Prepared Protein Structure Analysis

**Figure 2:**
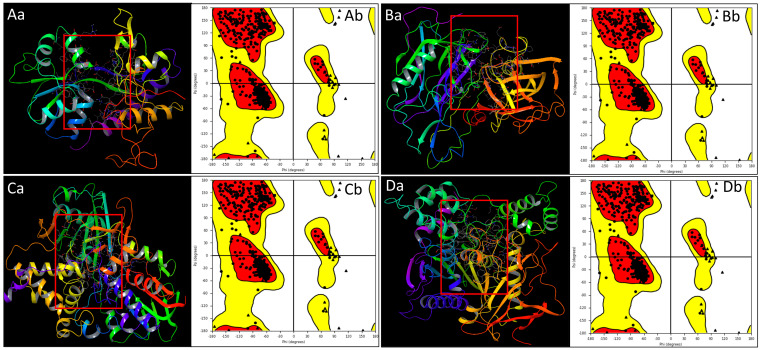
Three-dimensional structures of the prepared Alzheimer’s disease-associated proteins are processed using Maestro’s Protein Preparation Workflow (PPW). Panels (**Aa**–**Da**) depict the protein structures, with the ligand binding sites highlighted in red borders: (**Aa**) Human Monoamine Oxidase A (PDB ID: 2Z5X), (**Ba**) Human Acetylcholinesterase (PDB ID: 4EY6), (**Ca**) NR1 ligand-binding core (PDB ID: 1PBQ), and (**Da**) Human Beta Secretase 1 (PDB ID: 4D89). Corresponding Ramachandran plots validating the stereochemical quality of the prepared proteins are shown in panels for (**Ab**) 2Z5X, (**Bb**) 4EY6, (**Cb**) 1PBQ, and (**Db**) 4D89.

The PDB structures associated with Alzheimer’s disease—NR1 ligand-binding core (PDB ID: 1PBQ), Human Beta Secretase (BACE1, PDB ID: 4D89), Human Monoamine Oxidase A bound to Harmine (MAO-A, PDB ID: 2Z5X), and Human Acetylcholinesterase (AChE, PDB ID: 4EY6)—were prepared using the Protein Preparation Workflow to ensure proper structural configuration and readiness for subsequent analyses, thereby enhancing the reliability of this study’s results [23,25,27,33,37]. The structural irregularities, missing atoms, or unfavourable geometries in the protein molecules were fixed, which is essential to guarantee that the proteins under investigation are in a biologically relevant conformation, minimising any potential inaccuracies in our subsequent analyses and generating the Ramachandran plots for each protein to study the structural quality. The Ramachandran plot is a graphical representation that depicts the distribution of phi (ϕ) and psi (ψ) angles of amino acid residues in a protein structure. This analysis provides insights into the conformational quality of the protein and highlights regions that conform to energetically favourable configurations. Our detailed analysis of the Ramachandran plots for all four proteins revealed highly favourable outcomes. The majority of amino acid residues in each protein were positioned within the most favoured regions of the Ramachandran plot. The presence of amino acid residues in disallowed regions of the Ramachandran plot was minimal across all four proteins. These deviations were primarily located within loop regions, where a higher degree of flexibility is expected. The positive results obtained from the Ramachandran plot analysis underscore the success of our protein preparation efforts. The high conformational quality of the proteins established a solid foundation for subsequent analyses, including multitargeted docking and molecular dynamics simulations (Figure 2).

### 2.2. Molecular Interaction Studies

**Figure 3:**
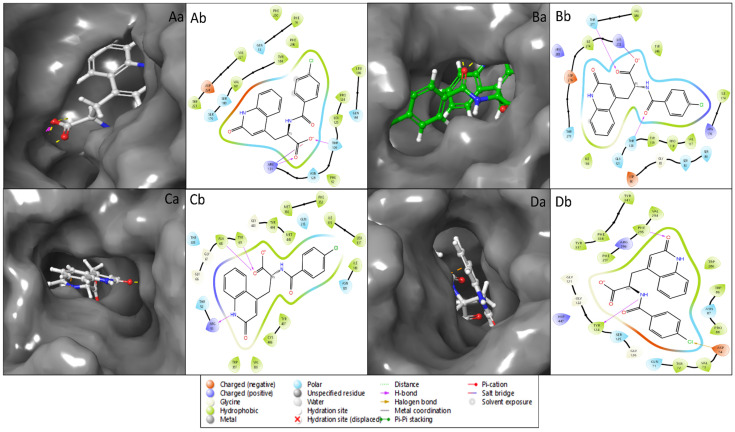
Protein–ligand interaction analysis of the four Alzheimer’s disease-associated target proteins for 3D interaction diagrams of the ligand binding sites for (**Aa**) Human Monoamine Oxidase A (PDB ID: 2Z5X), (**Ba**) Human Acetylcholinesterase (PDB ID: 4EY6), (**Ca**) NR1 ligand-binding core (PDB ID: 1PBQ), and (**Da**) Human Beta Secretase 1 (PDB ID: 4D89). In the 3D visualisations, ligands are depicted as stick models, while surrounding amino acid residues are shown as surface representations. Panels (**Ab**–**Db**) present corresponding 2D interaction diagrams: (**Ab**) 2Z5X, (**Bb**) 4EY6, (**Cb**) 1PBQ, and (**Db**) 4D89, illustrating hydrogen bonds, hydrophobic interactions, π-π stacking, and other relevant contacts between the ligand and active site residues. The interaction types and residue categories are indicated in the provided legend. All 2D and 3D ligand interaction diagram visualisations were generated using Schrödinger Maestro.

Molecular docking is a computational technique that predicts a small molecule’s binding mechanism and affinity to the protein targets that may be used to find new therapeutic candidates and has become a multidisciplinary approach to drug discovery that uses computational methods to improve the efficiency and effectiveness of the process and can be used to screen large libraries of compounds to accelerate drug discovery. Induced-fit docking studies examined the interactions between the ligands and flexible protein structures. Our study has identified Rebamipide as a multitargeted drug candidate against AD proteins [38,39]. In the case of Rebamipide and NR1 ligand-binding core (1PBQ), the docking score was found to be −9.32 Kcal/mol, and the MMGBSA score was −50.07 Kcal/mol (Table 1). The interaction between the ligand and the protein involved two hydrogen bonds, where the ligand formed hydrogen bonds with THR126 and ARG131 residues with two O atoms of the ligand and one salt bridge was also formed by an O atom with ARG131 residue (Figure 3Aa,Ab). Interaction with Human Monoamine Oxidase A (MAO-A) (2Z5X) showed a docking score of −11.02 Kcal/mol and MMGBSA score of −14.25 Kcal/mol (Table 1), with 3H bonds along ALA68 and TYR69 residues by O atoms and ARG51 residue interacting with the NH atom of the ligand Rebamipide (Figure 3Ba,Bb). Recombinant Human Acetylcholinesterase (AChE) (4EY6) produced a docking score of −10.73 Kcal/mol and MMGBSA score of −24.60 Kcal/mol (Table 1), and 2H bonding interacted with TYR124 residue by an NH atom and PHE295 residue by an O atom. Additionally, halogen bond contacts were made with ASP74 residue by the Cl atom of the ligand Rebamipide (Figure 3Ca,Cb). Rebamipide with Human Beta Secretase (BACE1) (4D89) produced a docking score of −4.58 Kcal/mol and MMGBSA score of −34.97 Kcal/mol (Table 1), with hydrogen bonding along THR120 and THR377 residues by two O atoms while a salt bridge contact was made between LYS272 residue and an O atom of the ligand (Figure 3Da,Db).

### 2.3. Pharmacokinetics Studies and Interaction Fingerprinting

The computed properties of Rebamipide using the QikProp tool were evaluated against standard values, revealing a comprehensive profile that underscores its potential as a drug candidate. Rebamipide exhibited favourable results across a spectrum of parameters, including QPlogS (−5.575), CIQPlogS (−5.164), QPlogHERG (−4.89), and QPlogKp (−3.625), all of which fell within the specified acceptable ranges. Notably, Rebamipide demonstrated properties such as CNS activity (−2), QPlogBB (−1.813), and skin permeation potential (QPlogKhsa 0.082) that align with desired criteria. Additionally, molecular features were in harmony with anticipated values, with indicators like #amidine, #amine, and #amide all registering 0. Rebamipide’s adherence to critical rules, including RuleOfThree and RuleOfFive, further enhanced its candidacy—the calculated presence of potential metabolic pathways (#metab 2) and compatibility with human oral absorption (2) added to its allure. Furthermore, the compound’s molecular characteristics, including QPlogPw (13.562), QPlogPC16 (13.867), QPlogPoct (21.294), and others, were all well within their respective ranges. It also encompassed attributes related to polarisability, hydrogen bonding, and molecular surface area, reinforcing its potential for drug-likeness. Impressively, Rebamipide’s properties for PercentHumanOralAbsorption (72.416), PSA (123.474), FISA (208.59), PISA (357.365), mol MW (370.791), SASA (672.933), volume (1144.025) collectively support Rebamipide as a promising candidate for further exploration in drug development endeavours (Table 2).

**Figure 4:**
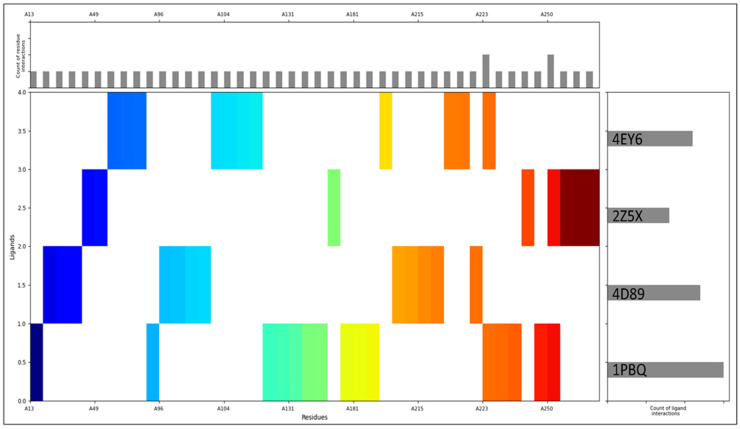
Molecular interaction fingerprints of Rebamipide–proteins complexes, where the ligand count and protein residue count are shown for better understanding.

The molecular interaction fingerprints were generated from the protein–ligand complexes after aligning them. The highest count of ligand interactions was found in 1PBQ, followed by 4D89, 4EY6, and 2Z5X, shown on the right side of Figure 4. The most residual interactions are shown in the upper histogram of Figure 4, and the main, coloured Figure shows the N to C terminal of the proteins. The interactions between Rebamipide and the target proteins were underpinned by specific amino acid residues, notably 3ALA, 2ARG, 3ASN, 4ASP, 2GLN, 1GLU, 2GLY, 3ILE, 4LEU, 3LYS, 1MET, 5PHE, 2PRO, 3SER, 1THR, 3TRP, 1TYR, and 2VAL. These interactions were integral to the stability of the Rebamipide–protein complexes, guided by various bonding forces like hydrogen bonding, electrostatic interactions, van der Waals forces, and hydrophobic interactions. Such diversity in residue types highlighted the intricate binding interactions that govern the complex stability. The stability of the complexes played a decisive role in determining the compound’s binding affinity, specificity, orientation within the protein’s binding site, and overall pharmacological impact. The range of implicated residues also sheds light on the compound’s distinct features, facilitating binding and potential therapeutic outcomes. Noteworthy interactions with hydrophobic residues implied that Rebamipide’s hydrophobic moieties contributed to binding, while interactions with polar residues suggested hydrogen bonding capabilities within the compound. Interactions involving charged and aromatic residues hinted at potential electrostatic and π-π interactions, further shaping binding and complex stability. The detailed characterisation of Rebamipide’s interactions with specific amino acid residues unveiled the intricate mechanisms that stabilised the complexes and unravelled the compound’s structural attributes, which enabled effective binding. These insights are vital for comprehending the molecular underpinnings of Rebamipide’s potential therapeutic effects in AD through a multitargeted approach.

### 2.4. WaterMap Analysis

**Figure 5:**
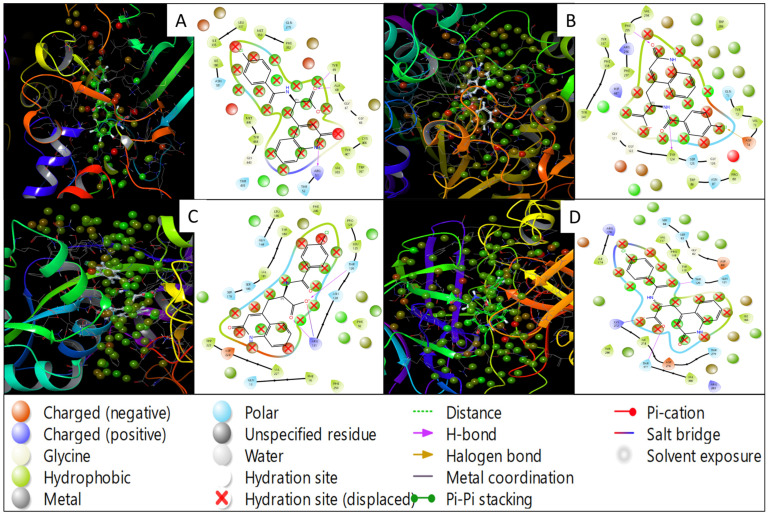
WaterMap results of Rebamipide in complex with PDBIDs with (**A**) 2Z5X, (**B**) 4EY6, (**C**) 1PBQ, and (**D**) 4D89.

The WaterMap analysis evaluated water thermodynamics at the binding sites of various protein–ligand complexes involving Rebamipide, focusing on three water-to-ligand transition states (trans1, trans2, and trans3) for each complex. The analysis highlighted changes in water entropy and enthalpy upon ligand binding. In the 2Z5X–Rebamipide complex, transition state 1 exhibited an energy change of −2.101 Kcal/mol, indicating favourable entropic gain from water displacement. Transition state 2 showed an energy change of 1.586 Kcal/mol, suggesting an unfavourable thermodynamic environment, while transition state 3 displayed an energy change of −4.070 Kcal/mol, highlighting a strong entropic contribution to binding affinity (Figure 5A). The 4EY6–Rebamipide complex revealed that transition state 1 had a substantial positive energy change of 8.248 Kcal/mol, indicating unfavourable binding due to inefficient water displacement. Transition state 2 exhibited an energy change of −5.848 Kcal/mol, suggesting significant entropic gain and enhanced binding affinity. In contrast, transition state 3 showed an energy change of 6.487 Kcal/mol, reflecting inefficient water displacement and an overall unfavourable binding effect (Figure 5B). For the 1PBQ–Rebamipide complex, transition state 1 had an energy change of −3.108 Kcal/mol, implying favourable binding due to water displacement. Transition state 2 recorded an energy change of −4.524 Kcal/mol, indicating substantial entropic gain and enhanced binding affinity, while transition state 3 had a slight negative energy change of −0.357 Kcal/mol, showing a modest positive impact on binding (Figure 5C). In the 4D89–Rebamipide complex, transition state 1 exhibited a high positive energy change of 8.534 Kcal/mol, suggesting that water displacement by Rebamipide was energetically unfavourable. Transition state 2 recorded an energy change of −5.356 Kcal/mol, indicating a strong entropic contribution to binding, whereas transition state 3 displayed an energy change of 2.575 Kcal/mol, highlighting an unfavourable interaction due to inefficient water displacement (Figure 5D). The WaterMap analysis highlights variations in water thermodynamics across different protein targets. While the 2Z5X–Rebamipide and 1PBQ–Rebamipide complexes exhibit favourable water displacement-enhancing binding affinity, the 4EY6–Rebamipide and 4D89–Rebamipide complexes show the critical role of water thermodynamics in drug binding efficacy.

### 2.5. DFT and TDDFT Analysis of Rebamipide

**Figure 6:**
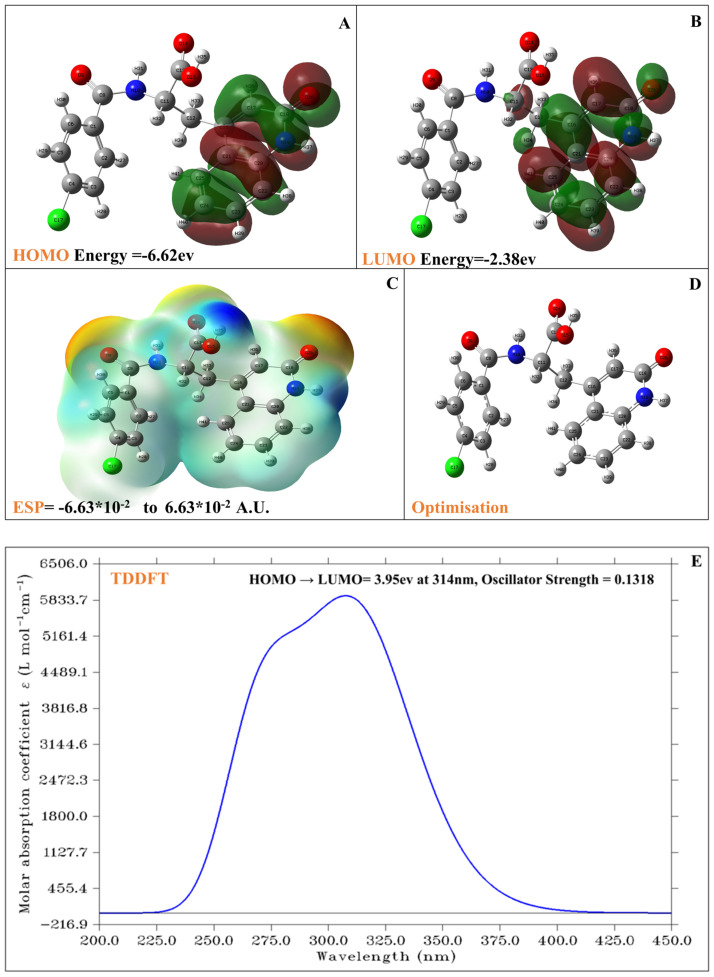
Showing the (**A**) HOMO Orbital Distribution—DFT-calculated electron density of the Highest Occupied Molecular Orbital (HOMO) at −6.62 eV, highlighting electron-rich regions. (**B**) LUMO Orbital Distribution—DFT-calculated LUMO orbital density at −2.38 eV, depicting potential electrophilic sites. (**C**) Electrostatic potential (ESP) surface—3D surface mapping the molecular ESP ranging from −6.63 × 10^−2^ to +6.63 × 10^−2^ A.U., indicating nucleophilic and electrophilic regions. (**D**) Optimised molecular structure—energy-minimised 3D geometry of Rebamipide using DFT methods. (**E**) TDDFT UV–Vis spectrum—simulated absorption spectrum showing a prominent transition at 314 nm (3.95 eV) with an oscillator strength of 0.1318, consistent with π→π* excitation.

The results of the density functional theory (DFT) computations performed using the Gaussian program provide a comprehensive analysis of the molecular properties of the compound. The compound Rebamipide, with the molecular formula C_19_H_15_C_l_N_2_O_4_, has a molecular weight of 370.79 g/mol and an exact mass of 370.0720347. It comprises 41 atoms, 4 hydrogen bond acceptors, and 3 hydrogen bond donors, with no formal charge. The compound exhibits a bioavailability score of 1, adheres to the Ghose filter, and complies with Lipinski’s Rule of Five, indicating favourable drug-like properties. It contains three rings and five rotatable bonds, with a polar surface area of 95.5 Å^2^, suggesting moderate polarity. The electronic structure of Rebamipide was analysed using density functional theory (DFT), providing detailed insights into its chemical reactivity and optical behaviour. The frontier molecular orbitals, namely the Highest Occupied Molecular Orbital (HOMO) and the Lowest Unoccupied Molecular Orbital (LUMO), are depicted in Figure 6A and Figure 6B, respectively. The HOMO energy of Rebamipide is calculated to be −6.62 eV, while the LUMO energy is −2.38 eV. This results in a HOMO-LUMO energy gap of 4.24 eV. Such a gap reflects the molecule’s kinetic stability and suggests moderate electronic reactivity. A large gap usually implies low reactivity and high stability, while a smaller gap indicates higher reactivity; hence, Rebamipide resides in an intermediate region, allowing for stable behaviour yet capable of participating in biologically relevant electron transfer reactions. The HOMO distribution (Figure 6A) is mainly delocalized over the electron-rich aromatic moieties and adjacent heteroatoms, particularly the oxygen and nitrogen atoms, indicating potential sites of electron donation. Conversely, the LUMO distribution (Figure 6B) highlights regions of electron acceptance, with electron density shifting toward the conjugated aromatic systems and electronegative atoms, making these regions likely candidates for electrophilic interactions. Figure 6C illustrates Rebamipide’s electrostatic potential (ESP) surface, offering a spatial understanding of charge distribution across the molecule. The ESP values range from −6.63 × 10^−2^ to +6.63 × 10^−2^ atomic units (A.U.), providing a symmetric bipolarity crucial for recognising potential interaction sites with biological targets. Regions shaded in red and orange correspond to high electron density, usually over oxygen and nitrogen atoms, suggesting nucleophilic centres suitable for hydrogen bonding or polar interactions. On the other hand, blue-coloured regions indicate electron-deficient zones, commonly over hydrogen atoms or less electronegative carbon environments, hinting at possible electrophilic interaction sites. The balanced distribution of ESP across the molecular surface underlines Rebamipide’s dual role in donor–acceptor chemistry, enhancing its pharmacophoric potential in biological systems. Figure 6D displays the optimised molecular geometry of Rebamipide, obtained through energy minimization under DFT constraints. The geometry confirms the planarity of the central aromatic core, which supports π-electron delocalization—an important feature for UV–Vis absorbance. Polar functional groups such as carboxyl, amide, and hydroxyl groups, all aligned in spatial proximity to the π-system, facilitate intramolecular charge transfer. These features contribute to molecular stability and enhance interaction capabilities with enzymes or receptors through hydrogen bonding or π-π stacking. Further insights into the optical properties of Rebamipide were obtained via Time-Dependent DFT (TDDFT) calculations, as shown in Figure 6E. The simulated UV–Vis absorption spectrum indicates a strong electronic transition from the HOMO to the LUMO, with an excitation energy of 3.95 eV, corresponding to a wavelength of 314 nm. This transition lies in the ultraviolet region, commonly associated with π → π* transitions within conjugated systems. The oscillator strength of this transition is 0.1318, reflecting a moderately allowed transition with reasonable intensity. The UV–Vis data extracted from the DATA_UV-VIS.txt file confirm the presence of this peak, showing a clear increase in molar absorptivity from 200 to 314 nm, with a maximum near the predicted transition. The sharp rise in absorbance in this region, followed by a gradual decline, indicates a single dominant transition without extensive overlap from other excited states, supporting the selectivity of Rebamipide’s electronic excitation behaviour. The molar absorption coefficient reaches a peak intensity of around 6500 L mol^−1^ cm^−1^, further validating the TDDFT prediction’s reliability.

### 2.6. Molecular Dynamics Simulation Studies

Molecular dynamics (MD) simulations were conducted for the four protein–Rebamipide complexes. These simulations provided valuable insights into the dynamic behaviour and stability of the protein–ligand complexes over time. The MD simulations involved subjecting the protein–ligand complexes to simulated environmental conditions, allowing the molecules to move and interact under the influence of physics-based forces. During the MD simulations, the structural stability of the complexes was assessed by monitoring parameters such as root mean square deviation (RMSD) and root mean square fluctuation (RMSF). The RMSD analysis evaluated the overall deviation of the complex structure from its initial configuration, providing insights into the stability of the interactions, whereas the RMSF analysis highlighted the flexibility of individual residues within the complex, indicating regions of higher mobility. Ultimately, the results from these MD simulations contribute to our knowledge of the multitargeted effects of Rebamipide in the context of AD and offer insights that can guide further research and drug development efforts. In the system builder, the Desmond package generated 39,452, 43,468, 71,297, and 55,319 atoms for 1PBQ, 4D89, 2Z5X, and 4EY6 in complex with Rebamipide, respectively, which were kept for the production until 100 ns, and the detailed analysis for deviation, fluctuations, and intermolecular interaction during simulations is as follows.

#### 2.6.1. Root Mean Square Deviation

**Figure 7:**
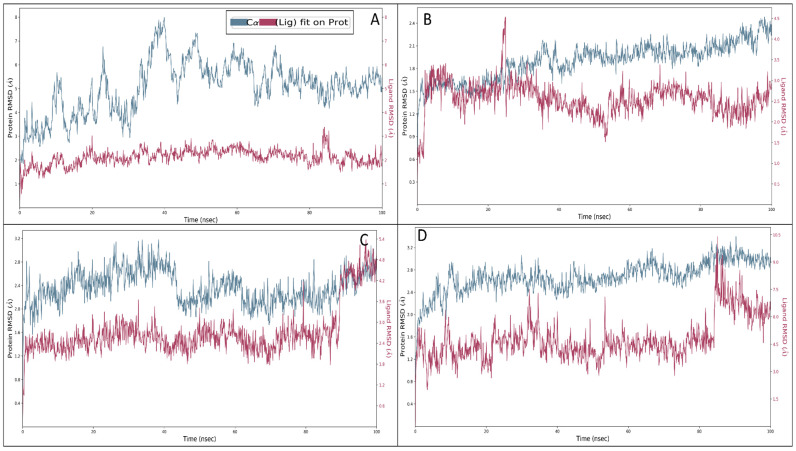
Root mean square deviation (RMSD) of Rebamipide–protein complexes, where red shows Rebamipide’s deviations in complex with (**A**) 2Z5X, (**B**) 4EY6, (**C**) 1PBQ, and (**D**) 4D89 in blue.

The root mean square deviations (RMSDs) become crucial to assessing compounds’ intricacy and steadfastness throughout the simulation. This metric quantifies the variances exhibited by proteins and ligands on a nanosecond scale, affording a comprehensive analysis at the molecular dimension. In the context of the Rebamipide and NR1 ligand-binding core (1PBQ) complex, initial minor oscillations were discernible, with protein and ligand deviations of 1.07 Å and 1.17 Å, correspondingly, at 0.10 ns. Nonetheless, as the simulation unfolded, the complex exhibited a trend of stability and at 100 ns, the protein deviation increased to 2.70 Å, and a corresponding ligand deviation of 4.67 Å was recorded. Nevertheless, except for the initial 1 ns interval, the complex showcased enduring stability (Figure 7A). In the Human Monoamine Oxidase-A (MAO-A) (2Z5X) complex with Rebamipide, a parallel sequence of initial deviation ensued, ultimately stabilising. At 0.10 ns into the simulation, the protein and ligand displayed divergences of 1.40 Å and 1.33 Å, respectively. After 100 ns of simulation, the protein deviation reached 4.85 Å, while the ligand divergence was noted to be 1.74 Å (Figure 7B), and if the initial deviation can be ignored, the complex showed a tremendous performance, which was the result of the initial heat, change in the solute medium, and ensemble class in neutralising it. The Human Acetylcholinesterase (AChE) (4EY6) complex with Rebamipide underwent initial deviations, and at 0.10 ns, the protein and ligand deviated to 1.03 Å and 1.25 Å, respectively. As the simulation kept going over 100 ns, the protein and the ligand displayed significantly fewer deviations, quantified at 2.24 Å and 2.80 Å, which is acceptable for the protein–ligand complex (Figure 7C). The Human Beta Secretase (BACE1) (4D89) in complex with Rebamipide demonstrated an initial deviation of 1.23 Å and 2.46 Å at 0.10 ns for protein and ligand, respectively. Nevertheless, at 100 ns of simulation, the protein deviation escalated to 3.02 Å, whereas it went to 6.82 Å for the ligand, which might result from initial heat, ensemble class, change in solute medium, and added ions to neutralise the system for production. In keeping with the pattern observed in the preceding complexes, except for the first, stability characterised the complex’s performance throughout the simulation (Figure 7D), which is acceptable for the biological complexes. The deviation studies for all the complexes seem acceptable and suggest that the complexes are stable in the experimental studies.

#### 2.6.2. Root Mean Square Fluctuations

**Figure 8:**
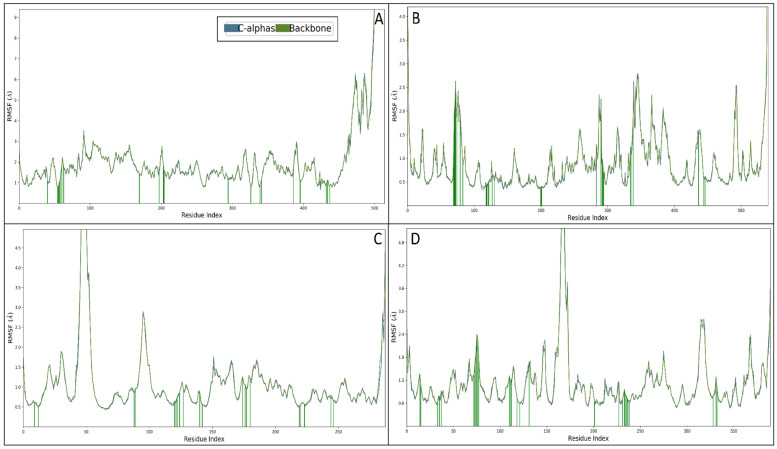
The root mean square fluctuation (RMSF) proteins are shown in blue for (**A**) 2Z5X, (**B**) 4EY6, (**C**) 1PBQ, and (**D**) 4D89, and the green line shows the interactions of protein residues with Rebamipide to make the complex stable.

The root mean square fluctuation (RMSF) values were computed for the protein structures complexed with Rebamipide to evaluate their flexibility and interaction stability. RMSF is a useful metric for understanding the local dynamic behaviour of amino acid residues in a protein–ligand complex. Higher RMSF values indicate flexible regions, while lower values suggest stable interactions. The RMSF analysis of the NR1 ligand-binding core complexed with Rebamipide revealed several fluctuating residues beyond 2 Å, including GLU58, HIS59, PRO72, SER94, GLU95, TYR100-GLU137, and ALA146-GLY213. These fluctuations suggest localised flexibility within these regions, likely due to conformational changes upon ligand binding. The interacting residues identified were ARG51, VAL65, GLY66-GLN74, ILE180, PHE208, GLY214, GLN215, LYS305, LEU337, MET350, PHE352, TRP397, CYS406, TYR407, GLY443-MET445, and ALA448, which contribute to ligand stabilisation and complex integrity. The presence of key interactions within these residues highlights their role in Rebamipide binding and potential functional implications (Figure 8A). The RMSF profile of the Human Beta Secretase complex indicated fluctuating residues beyond 2 Å, including GLU4, THR75-GLU81, PRO290, SER293, GLY342, GLY345-GLU351, GLN369, LEU386, PRO495, LYS496, and LEU539-ALA542. These fluctuations suggest intrinsic flexibility in these regions, which may influence ligand binding and enzymatic function. Notably, the interacting residues TYR72-THR75, TYR77, GLY82, TRP86, GLY121, GLY122, TYR124, SER125, LEU130, TYR133, GLU202, SER203, ALA204, TRP286, SER293-PHE297, TYR337, PHE338, TYR341, TRP439, HIS447, and TYR449 were crucial in stabilising the ligand within the active site, ensuring proper interaction and structural coherence (Figure 8B). For the Human Monoamine Oxidase A complex, fluctuating residues beyond 2 Å were observed in ASN48-HIS57, VAL98-SER101, GLU289, and CYS290. These residues exhibited significant movement, likely due to induced fit mechanisms upon ligand binding. The interacting residues identified, including GLN13, PHE16, PHE92, GLY93, PRO124, LEU125, THR126, ASN128, ARG131, GLN144, LEU146, GLN178, SER180, VAL181, TYR184, TRP223, ASP224, VAL227, SER248, and PHE250, were found to be critical for maintaining complex stability. The presence of aromatic and hydrophobic residues within the interacting site suggests a stable binding conformation facilitated by hydrophobic and π-π interactions (Figure 8C). In the Human Acetylcholinesterase complex, fluctuating residues beyond 2 Å were identified as VAL48, THR120, GLN121, THR192, HIS193, PRO208-VAL218, ASP359-GLN364, GLU412, and PHE413. These flexible regions might be involved in substrate recognition and conformational adjustments upon ligand binding. The interacting residues GLY59, GLN60, LEU78, ASP80, GLY82, VAL117, PRO118, TYR119, THR120, GLN121, GLY122, LYS155, PHE156, PHE157, TRP163, ILE166, ARG176, TYR246, LYS272, ASP276, GLY278, THR279, THR280, ASN281, ARG283, SER373, SER376, and THR377 played a significant role in ligand stabilisation. The presence of polar and charged residues among these interactions suggests the contribution of hydrogen bonding and electrostatic forces in maintaining complex stability (Figure 8D). The RMSF analysis provides valuable insights into the dynamic behaviour of Rebamipide when bound to different target proteins. Regions with high fluctuation suggest flexible binding pockets or allosteric sites that may be crucial for ligand accommodation and conformational changes. Conversely, stable interacting residues indicate strong binding sites contributing to the ligand’s efficacy. Across all complexes, key interactions involve a combination of hydrogen bonds, hydrophobic forces, and electrostatic interactions, emphasising the multifaceted nature of ligand binding. The presence of aromatic residues such as PHE, TYR, and TRP in several interactions suggests that π-π stacking plays a role in Rebamipide binding. Additionally, charged residues such as ARG, LYS, and GLU contribute to electrostatic interactions that further stabilise the complex. The findings from this RMSF analysis highlight the importance of flexible and stable regions in ligand binding, providing a foundation for further computational or experimental validation. These results suggest that Rebamipide forms stable interactions with the studied proteins, making it a promising candidate for further drug design and optimisation efforts.

#### 2.6.3. Simulation Interaction Diagram

**Figure 9:**
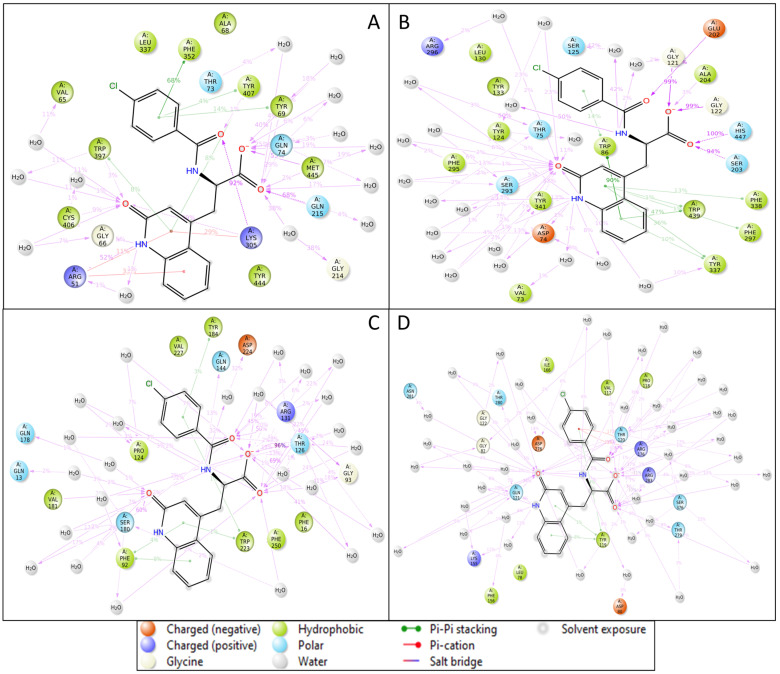
The simulation interaction diagram of the complex of Rebamipide with (**A**) 2Z5X, (**B**) 4EY6, (**C**) 1PBQ, and (**D**) 4D89 and legend are provided to understand residue and bond types.

**Figure 10:**
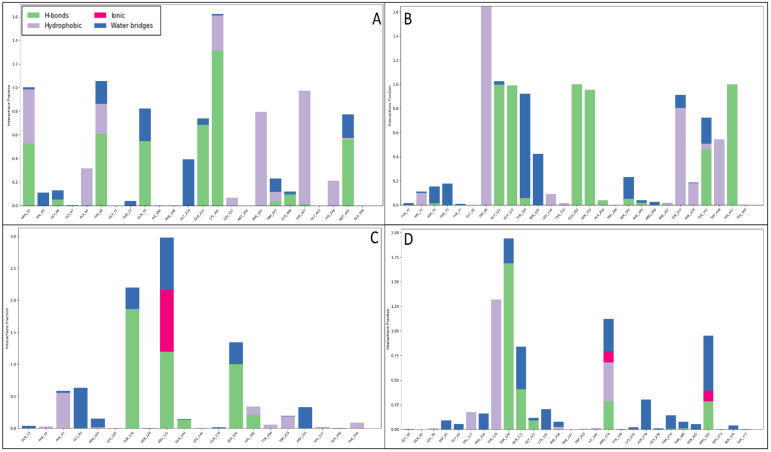
The count of interactions during simulation between the Rebamipide and (**A**) 2Z5X, (**B**) 4EY6, (**C**) 1PBQ, and (**D**) 4D89, where green shows the hydrogen bonds, red shows the ionic, grey shows hydrophobic interactions, and blue shows the water bridges.

Throughout the MD simulations, the interaction between the ligand and protein generated a Simulative Interaction Diagram (SID) for each complex, highlighting key stabilising forces. Various molecular interactions, such as hydrogen bonding, water-bridging networks, π-π stacking, π–cation contacts, and salt bridges, played a crucial role in stabilising the ligand within the active sites of target proteins. In the case of the NR1 ligand-binding core (1PBQ) complex with Rebamipide, extensive water-mediated stability was observed, with 15 water molecules forming water bridges. The ligand established multiple hydrogen bonds, particularly ARG131, THR126, SER180, and VAL181 residues through O atoms and GLN144 and THR126 through NH atoms. Additionally, the benzene ring of the ligand engaged in π-π stacking interactions with PHE92 and TYR184, further contributing to ligand stabilisation. These interactions enhance ligand specificity, ensure stable positioning within the binding pocket, and strengthen intermolecular contacts through water bridges, which act as additional stabilisation networks. The π-π stacking between aromatic residues and the benzene ring increases binding affinity by stabilising the ligand orientation (Figure 9A). For the Human Monoamine Oxidase A (MAO-A) (2Z5X) complex with Rebamipide, the interaction was water-dominated, with over 10 water molecules forming stabilising bridges. Extensive hydrogen bonding was observed with TYR407, LYS305, TYR69, GLN74, GLN215, and TRP397, interacting through four O atoms and NH atoms. Additionally, GLY66 and ARG51 residues contributed to NH-mediated hydrogen bonding. Notably, π–cation interactions were observed between LYS305 and ARG51 residues with the benzene ring, while π-π stacking interactions involved PHE352, TYR407, TYR69, and TRP397. Π–cation interactions between positively charged residues such as ARG and LYS and the ligand’s benzene ring contribute to enhanced electrostatic stability, while multiple hydrogen bonds increase ligand binding specificity and affinity. π-π stacking ensures proper ligand orientation within the hydrophobic pocket of MAO-A, further reinforcing its stability (Figure 9B). The interaction between Rebamipide and Human Acetylcholinesterase (AChE) (4EY6) resulted in extensive hydrogen bonding with GLU202, GLY121, ALA204, GLY122, HIS447, SER203, SER293, and TYR341, engaging via four O atoms. Additionally, ASP74 and TYR341 residues contributed to water-mediated NH interactions. Many π-π stacking interactions were identified with TYR337, TRP439, PHE297, PHE338, and TRP86, facilitating ligand stabilisation within the binding pocket. Moreover, a π–cation interaction was observed with HIS418, and a salt bridge interaction involving ASP325 and the N^+^H_3_ atom further enhanced stability. Salt bridges provide additional electrostatic attraction, significantly contributing to ligand stability. π-π stacking ensures proper ligand orientation within AChE’s active site, while hydrogen bonding enhances specificity and ligand retention, allowing for strong interaction stability throughout the simulation (Figure 9C). In the case of the Human Beta Secretase (BACE1) (4D89) complex with Rebamipide, an extensive network of interactions was observed, with over 156 water bridges, highlighting the importance of hydration in ligand stabilisation. Hydrogen bonds were observed with THR120, ARG176, ARG283, THR279, GLU146, ARG35, and GLN121 residues, interacting through three O atoms and the N^+^H_3_ atom. Additionally, interactions involving LYS200, GLU98, LEU145, VAL147, ARG35, and GLN121 occurred via NH atoms, while PRO143 residues utilised three OH atoms. The ligand also formed π-π stacking interactions with TYR119 and π-cation interactions with ARG176 and ARG35, further enhancing binding stability. Extensive water bridges suggest that hydration plays a critical role in ligand stability, allowing for stronger hydrogen bonding networks and increased ligand retention within the active site. Multiple hydrogen bonds contribute to specificity and affinity within the active site, while π-π stacking and π–cation interactions enhance ligand retention by stabilising aromatic interactions (Figure 9D). The stability of protein–ligand complexes is primarily dictated by noncovalent interactions, which govern molecular recognition, specificity, and affinity. Hydrogen bonds act as key stabilising forces by bridging polar residues and ligand functional groups, ensuring ligand positioning within hydrophilic active sites, and being reinforced by solvent molecules through water bridges. Water bridges mediate interactions between the ligand and distant protein residues, strengthening ligand binding, which is particularly significant in highly hydrated enzyme pockets such as BACE1. π-π stacking interactions between aromatic amino acids such as PHE, TYR, and TRP and benzene rings in the ligand are essential for ligand stabilisation in hydrophobic binding sites, as seen in the MAO-A and AChE complexes. Π–cation interactions occur between positively charged residues such as ARG, LYS, and HIS and the ligand’s aromatic system, providing additional electrostatic stabilisation, crucial in neurotransmitter-binding proteins like MAO-A and AChE. Salt bridges, formed between oppositely charged residues and ligand functional groups such as ASP-ARG interactions, provide strong electrostatic interactions, increasing ligand retention in the binding pocket and contributing significantly to binding affinity. Additionally, the histogram shown in Figure 10 provides a quantitative visualisation of the interaction counts formed during MD simulations, further validating the significance of these noncovalent interactions in complex stabilisation.

## 3. Discussion

Among the drugs screened, Rebamipide, a neuroprotective agent used in gastrointestinal disorders, exhibited notable multitargeted binding interactions with all four AD-related proteins. Our results suggest that Rebamipide has the potential to act as a multitargeted inhibitor, impacting multiple aspects of AD pathology. Further, we performed MD simulations for each protein–ligand complex to validate the stability of these interactions. The MD simulations provided crucial insights into the dynamic behaviour of the Rebamipide–protein complexes, confirming their robustness and stability over time. Moreover, our analysis of intermolecular interactions sheds light on Rebamipide binding mechanisms and potential functional significance in each protein complex. Identifying Rebamipide as a multitargeted drug candidate against AD offers a promising direction in searching for effective therapeutic options for this devastating disease. Its potential to interact simultaneously with essential proteins involved in AD pathogenesis makes it a compelling candidate for further experimental investigation and preclinical studies. Multitargeted approaches like this hold great promise in addressing the complexity of AD and may pave the way for developing novel and effective treatments for this challenging condition. The interconnected roles of NR1 ligand-binding core, BACE1, MAO-A, and AChE in AD pathogenesis underscore the complexity of the disease [23,25,27,33,37]. Targeting multiple proteins simultaneously with multitargeted drugs like Rebamipide offers a promising approach to addressing the multifactorial nature of AD and holds significant potential for developing more effective therapeutic interventions against this devastating neurodegenerative disorder [8,9,16]. Further research and experimental validation are warranted to explore the clinical utility of Rebamipide as a multitargeted compound for AD.

The preparation of protein structures played a pivotal role in ensuring the reliability and accuracy of subsequent analyses where we rectified structural irregularities, missing atoms, and unfavourable geometries of NR1 ligand binding core (1PBQ), Human Beta Secretase (BACE1) (4D89), the Human Monoamine Oxidase A (MAO-A) complex with Harmine (2Z5X), and Human Acetylcholinesterase (AChE) (4EY6) proteins and validated with Ramachandran plots [23,25,27,33,37]. The molecular interaction studies’ results showcased Rebamipide’s potential as a multitargeted drug candidate against AD, which involved hydrogen bonds, salt bridges, pi–cation interactions, and benzene interactions with various amino acid residues. Notably, each protein–ligand complex exhibited distinct interaction patterns, suggesting that Rebamipide’s multifaceted interactions contribute to its potential as a multitargeted drug. Pharmacokinetic studies utilising the QikProp tool comprehensively assessed Rebamipide’s properties against standard values. Rebamipide demonstrated compatibility with various parameters, such as QPlogS, QPlogHERG, and QPlogKp, aligning well with the desired criteria. The compound’s adherence to critical rules, such as RuleOfThree and RuleOfFive, highlighted its drug-likeness. The diversity of interacting residues, including hydrophobic, polar, charged, and aromatic, reflected Rebamipide’s multifaceted binding interactions that shaped the compound’s binding affinity, specificity, orientation within the protein’s binding site, and overall pharmacological impact. The DFT results provide valuable insights into the electronic structure and properties of the compound, highlighting its drug-like characteristics. The HOMO-LUMO energy gap suggests moderate reactivity, while the solvation energy indicates favourable interactions in aqueous environments. The compound’s lipophilicity (logP = 2.76) and polar surface area (95.5 Å^2^) align with drug-likeness criteria. Electrostatic potential and ALIE analyses reveal the charge distribution and reactive sites, aiding in understanding molecular interactions. Rebamipide’s DFT and TDDFT analyses highlight a well-balanced electronic architecture featuring moderate reactivity, optimal charge distribution, and stable geometry, which collectively support its bioactive potential. The strong π-conjugation and distinct HOMO-LUMO character suggest good capability for interacting with biological macromolecules, while the predicted absorption in the UV region confirms electronic delocalization supportive of antioxidant or anti-inflammatory activities. These findings corroborate the compound’s pharmacological relevance and provide a foundational understanding for further studies, including docking, MD simulations, and experimental validations. The stabilisation of these complexes played a decisive role in Rebamipide’s potential therapeutic effects in AD, underscoring its multitargeted approach [8,9,16]. The MD simulation studies further expanded our understanding of Rebamipide’s behaviour within the protein–ligand complexes. The root mean square deviation (RMSD) and root mean square fluctuation (RMSF) analyses revealed that the complexes maintained acceptable stability throughout the simulations. The RMSD profiles indicated initial minor oscillations followed by overall stability, and the RMSF analysis highlighted specific residues with varying flexibility. These simulation results affirmed the stability, contributing crucial insights for multitargeted drug candidates. The molecular interactions observed in these protein–ligand complexes highlight the multifaceted nature of ligand stabilisation, where hydrogen bonding, water bridges, π-π stacking, π–cation contacts, and salt bridges collectively contribute to Rebamipide’s binding strength and specificity. Each interaction type plays a distinct role in reinforcing ligand retention, improving structural stability, and enhancing binding efficiency within the protein active sites. Understanding these interactions is crucial for rational drug design, enabling the modification and optimisation of drug candidates to achieve enhanced therapeutic efficacy [38,39].

## 4. Materials and Methods

We have drawn Figure 1 as the workflow to make this study concisely clear to understand, and the detailed methods are as follows.

### 4.1. Data Collection, Preparation, and Secondary Structure Analysis

We used the RCSB-PDB (https://rcsb.org/) (accessed on 30 May 2024) database for the 3D crystal structures of proteins and found NR1 ligand binding core (PDBID: 1PBQ), Human Beta Secretase (PDBID: 4D89), Human Monoamine Oxidase A with Harmine (PDBID: 2Z5X) and Human Acetylcholinesterase (PDBID: 4EY6), which actively participate in AD [23,25,27,33,37,40,41]. In 1PBQ, Chain A, Chain B, solvents, and ligands were found, in 2Z5X, Chain A with solvents and ligands were found, in 4EY6, we found Chain A and Chain B with solvents and ligands, along with the other metals/ions, and in 4D89, we found Chain A with ligands. For protein preparation, we used Maestro’s Protein Preparation Workflow (PPW) tool, where in preprocess, we filled in the side chains, capped termini, assigned bond orders to the Chemical Component Dictionary (CCD) database, replaced hydrogens and created disulphide bonds, converted selenomethionines to methionine, filled in missing loops using Prime, and generated heterostates with Epik at pH 7.4 ± 2 [42,43,44,45]. Sample water orientation and crystal symmetry were used for optimisation, and minimised water of altered species and PROPKA (Protein pKa) optimisation were used. Further, in the minimisation of proteins, heavy atoms were converged to RMSD 0.30 Å with the OPLS4 forcefield for minimisation after deleting water beyond 5 Å from heteroatoms that could potentially create any interaction conflicts [46,47,48,49,50,51,52]. The same parameters were used for the PPW in each condition, and after preparations, only Chain A was kept in 2Z5X, 4EY6, and 1PBQ, and Chain A with ligands was kept in 4D89, and on the same file we generated the grids. For the ligand library, we used the FDA-approved ligand library from the Selleckchem (https://www.selleckchem.com/) (accessed on 30 May 2024) database and prepared with the LigPrep tool Maestro (https://www.schrodinger.com/) (accessed on 30 May 2024), where we kept the filter of 500 atoms with the OPLS4 forcefield and generated the possible states at the target pH 7 ± 2, and Epik was used in ionisation [42,52,53,54]. Desalt and tautomer generation and stereoisomer computations were kept, retaining specified chiralities and generating at most 32 per ligand.

### 4.2. Receptor Grid Generation and Molecular Interaction Analysis

The Receptor Grid Generation tool in Maestro (https://www.schrodinger.com/) (accessed on 30 May 2024) was used to generate grids on the proteins’ active site, which is a crucial step before molecular docking to make the software understand where to make the calculations and reduce the computational costs [55]. PDBID 2Z5X has only Chain A, and we selected whole protein under the grid, whereas in PDBIDs 4EY6, 1PBQ, and 4D89 ligands were present, so we selected the ligand site as the active site in the receptor grid generation tool [23,25,27,33]. We picked the molecule for the cases of native ligands, and van der Waals radius scaling at a scaling factor of 1.0 and a partial charge cutoff of 0.25 were kept, and the enclosing box was selected as the centroid of the workspace ligand, and then we adjusted the size of the box to cover most of the active site, and rest of the parameters were kept at default. For the docking studies, we used the Virtual Screening Workflow (VSW) tool, where, in the input tab, we browsed the FDA-approved prepared drug library, combined the input file, and redistributed for subjects; unique compounds were selected and the duplicates were removed [42,56]. In the filtering tab, we kept the QikProp [57] filter to generate the ADMET properties and prefilter with Lipinski’s rule [58], which used the QikProp data, and the preparations tab was kept empty as the ligands were already prepared, and in the receptor tab, the grid file was browsed one by one to submit the screening jobs. In the docking tab, we use the Epik penalties [44] for docking and ligand van der Waals radii for nonpolar atoms with a scaling factor of 0.80 and partial charge cutoff of 0.15. Further, for the docking, we used all three available options of High-Throughput Virtual Screening (HTVS), Standard Precision (SP), and Extra Precise (XP) calculations followed by molecular mechanics with generalised Born and surface area solvation (MM\GBSA) studies [45]. In HTVS, 100% of data were selected and only the top 10% of data were passed to SP and, again, the top 10% of data were passed to XP for precise calculations, where we kept generating up to 4 poses per compound state, and after docking 100% of the data were passed to MMGBSA for binding free energy calculation.

### 4.3. Pharmacokinetics and Molecular Fingerprint Analysis

For the pharmacokinetic properties of the identified compound, we used the QikProp tool [57], which computes precisely the molecular weight, octanol–water partition coefficient (logP), aqueous solubility, polar surface area (PSA), number of hydrogen bond donors, number of hydrogen bond acceptors, number of rotatable bonds, number of rings, human intestinal absorption [25] prediction, blood–brain barrier (BBB) penetration prediction, plasma protein binding (PPB) prediction, caco-2 permeability prediction (cellular permeability), rat oral bioavailability prediction, human oral bioavailability prediction, metabolic stability prediction (T1/2), CYP450 inhibition predictions (CYP2C9, CYP2D6, and CYP3A4), quantitative estimate of drug-likeness (QED) score, Lipinski’s Rule of Five compliance, and prediction of human Ether-à-go-go-Related Gene (hERG) channel binding (cardiac safety). Further, the Interaction Fingerprints tool was used on protein–ligand complexes, where we selected any contact for maximum interaction outputs [42]. We aligned all complexes to make them look sequential, keeping 1PBQ as a reference, all advanced features as default, and generating the fingerprint. On the matrix, we coloured the main plot from the N to C terminal while involving any contact-type residues and kept only interacting residues to make it more straightforward.

### 4.4. Water Thermodynamics (WaterMap Studies)

WaterMap analysis is a computational technique used in drug design to evaluate the thermodynamic properties of water molecules at the binding sites of proteins. Analysing the displacement of water molecules upon ligand binding helps identify “hot spots” where water removal is energetically favourable, enhancing binding affinity. WaterMap calculates the enthalpic and entropic contributions of water molecules, guiding the optimisation of drug candidates. This analysis is crucial for understanding the role of water in ligand binding, improving the design of drugs with better efficacy and specificity by targeting key interactions at the molecular level. It is instrumental in refining binding sites for more potent and selective drug candidates. For WaterMap computations, we used the WaterMap- Perform calculation panel in Schrödinger Maestro (https://schrodinger.com/) (accessed on 30 May 2024), where we selected the ligand from the workspace in the P-L complexes and retained the ligand for proper computations. The water within 10 Å of the ligand was analysed, the OPLS4 force field treated the existing water as a solvent, and the MD simulation time was kept to 5 ns to extract better and more efficient results and not to return the trajectories [42,59]. Further, we used the WaterMap- Examine results to analyse the computed water thermodynamics, which analysed various energies and transitions along with the 2D diagrams to better understand each point [42,59]. Despite its advantages, WaterMap has limitations that must be considered while interpreting the results. One major constraint is its reliance on force field parameters, which may introduce inaccuracies in predicting water energetics, especially in highly flexible or polar binding sites. Additionally, the method assumes that water molecules sampled within the simulation time frame represent equilibrium hydration states, which might not always be the case. Longer simulations or enhanced sampling techniques, such as meta-dynamics, could provide a more comprehensive picture of hydration dynamics. Another important aspect is the potential for error propagation when integrating WaterMap with other computational methods. For instance, docking and free energy perturbation calculations depend on different assumptions and force field implementations, which may introduce inconsistencies when combined with WaterMap data. Thus, while our WaterMap results provide valuable qualitative insights into hydration site energetics, they should be interpreted cautiously when making quantitative predictions. Given these considerations, our WaterMap analysis serves primarily as a qualitative tool to refine binding site characterisation rather than a definitive predictor of absolute binding affinities. Future studies incorporating longer simulations and alternative solvation models could further improve the accuracy of hydration site predictions.

### 4.5. Density Functional Theory (DFT) and TDDFT Calculations

Density functional theory (DFT) plays a crucial role in drug design by offering quantum mechanical insights into compounds’ electronic structure and properties. It aids in understanding molecular interactions, reactivity, and stability, facilitating accurate predictions of ligand behaviour within biological systems. In multitargeted drug design, DFT is essential for assessing molecular orbitals, electron densities, and energy levels, which are vital for optimising drug candidates to enhance binding affinity and selectivity [60,61]. To explore the electronic properties and reactivity descriptors of the studied molecule Rebamipide, density functional theory (DFT) calculations were performed using the Gaussian 09 software package [62]. We employed the widely validated B3LYP hybrid functional, which combines Becke’s three-parameter exchange functional [63] with the Lee-Yang-Parr correlation functional [64]. This hybrid function offers a reliable balance between computational cost and accuracy and is extensively used to investigate electronic properties in organic and bioactive molecules. For the basis set, we selected 6-311++G(d,p), a triple-ζ split-valence basis set developed by Krishnan et al. [65] and later extended with diffuse and polarisation functions by Clark et al. [66]. The (d,p) polarisation functions help in better describing the anisotropy in electron density, particularly for heavier atoms and hydrogens, while the “++” diffuse functions allow accurate treatment of valence electrons and long-range interactions, which is crucial for excited-state and charge-transfer studies [67]. Together, this level of theory (B3LYP/6-311++G(d,p)) ensures a robust and accurate description of molecular geometries and electronic structures [68]. A complete geometry optimisation was conducted to locate the minimum-energy conformation of the molecule. A vibrational frequency analysis was performed to confirm that the structure corresponds to a true minimum on the potential energy surface; the absence of imaginary frequencies validated the nature of the stationary point [69]. Using the optimised geometry, the energies of the Highest Occupied Molecular Orbital (HOMO) and Lowest Unoccupied Molecular Orbital (LUMO) were computed [70,71]. These orbitals are central to understanding the chemical reactivity and stability of the molecule: the HOMO indicates the molecule’s capacity to donate electrons, whereas the LUMO indicates its ability to accept electrons. The energy gap (ΔE = ELUMO − EHOMO) was used to estimate the electronic hardness and reactivity. The molecular orbitals were visualised using GaussView software 6.0 [72]. The molecular electrostatic potential (ESP) surface was computed using the optimised geometry to evaluate the electronic charge distribution across the molecular surface. ESP mapping helps identify potential electrophilic and nucleophilic attack sites and understand intermolecular interactions. These surfaces were mapped onto the molecule’s van der Waals surface and visualised in GaussView [72]. Time-Dependent DFT (TD-DFT) calculations were performed at the same B3LYP/6-311++G(d,p) level to study the electronic excitation behaviour. This approach enables the prediction of vertical excitation energies and simulates the UV–visible absorption spectrum by computing oscillator strengths and transition wavelengths for several low-lying excited states [73,74]. The UV–Vis spectra were visualised using Multiwfn software 3.8 [75], and final plots were generated using Origin 8.0 [76].

### 4.6. System Builder and Molecular Dynamics Simulation Studies

We used molecular dynamics (MD) simulation methods to validate docked results using the Desmond package, developed by D. E. Shaw Research and available freely for academic use from https://www.deshawresearch.com/ (accessed on 30 May 2024) [42,77]. The MD simulation was conducted in two steps: preparing the model and conducting the production run. The System Builder panel was used to prepare the complete system, where we excluded ion and salt placement within 20 Å and added 19Cl^−^, 8Cl^−^, 29Cl^−^, and 32Cl^−^ in 1PBQ, 4D89, 2Z5X, and 4EY6 to neutralise the system. The SPC water model was used in orthorhombic conditions in buffer calculations of 10 × 10 × 10 Å and minimised the volume to fix the box entirely on the complex and used the OPLS4 forcefield [52,78]. Further, in the second step, we used the Molecular Dynamics panel for the production run. We loaded the system builder file in the panel and set the production time to 100 ns, with a recording interval of 100 ps with an energy level of 1.2, generating 1000 frames in each condition. The NPT ensemble class provided more accurate calculations for the P-L complexes at a 300 K temperature and 1.01325 bar pressure and relaxed the system before production to optimise it [79]. The simulation interaction diagram generated the EAF file to analyse the MD simulation results [42].

## 5. Conclusions

Our comprehensive study illuminates the potential of Rebamipide as a multitargeted drug candidate for AD treatment. We unveiled Rebamipide’s significant affinity and interactions with AD-related proteins, such as NR1 ligand binding core, Human Beta Secretase, Human Monoamine Oxidase A, and Human Acetylcholinesterase, through meticulous molecular interaction analyses. These interactions, driven by hydrogen bonds, salt bridges, pi–cation interactions, and benzene interactions, underscore Rebamipide’s capacity for multitargeted inhibition. Pharmacokinetic profiling confirmed favourable drug-likeness, while interaction fingerprinting showed consistent hydrogen bonding, hydrophobic contacts, and π-π stacking interactions across complexes. WaterMap analysis revealed thermodynamically favourable water displacement, enhancing ligand affinity. DFT calculations showed a HOMO-LUMO gap of 4.24 eV and an ESP range of −6.63 × 10^−2^ to +6.63 × 10^−2^ A.U., indicating both electronic stability and reactive binding regions. TDDFT analysis predicted strong UV absorbance at 314 nm with a peak molar absorptivity of ~6500 L mol^−1^ cm^−1^. Moreover, 100 ns MD simulations demonstrated minimal structural deviations, confirming stable and sustained protein–ligand interactions. These findings emphasise Rebamipide’s potential as a promising multitargeted drug candidate for Alzheimer’s disease therapy; however, experimental studies are needed before human use.

## Figures and Tables

**Table 1:** The docking score (Kcal/mol) and MM\GBSA (Kcal/mol) against each PDB.

S No	PDB	Docking Score	MMGBSA	Prime Hbond	Prime vdW
1	4EY6	−10.738	−24.6	−289.16	−2945.74
2	2Z5X	−11.022	−14.25	−305.76	−2747.19
3	4D89	−4.581	−34.97	−189.86	−1895.04
4	1PBQ	−9.321	−50.07	−152.29	−1403.84

Prime Hbond and Prime vdW represent components of the molecular mechanics-based energy decomposition calculated during the Prime MM\GBSA post-docking analysis in the Schrödinger Suite. Prime Hbond (Hydrogen Bond Energy): represents the electrostatic energy contribution from hydrogen bonding interactions between the ligand and protein. Prime vdW (van der Waals Energy): reflects the non-bonded van der Waals interactions between the ligand and protein atoms within proximity. Both values are expressed in kcal/mol and are calculated using the OPLS4 force field within the MM\GBSA (molecular mechanics–generalised Born surface area) framework implemented in the Prime module of Schrödinger Maestro. These energies help assess the strength and nature of the binding interactions beyond docking scores alone.

**Table 2:** Pharmacokinetic properties were computed with QikProp and compared with standard descriptors.

Properties	Standard Values	Rebamipide	Properties	Standard Values	Rebamipide
QPlogS	−6.5–0.5	−5.575	donorHB	0.0–6.0	2.25
CIQPlogS	−6.5–0.5	−5.164	QPlogPo/w	−2.0–6.5	3.421
QPlogHERG	concern below −5	−4.89	#rotor	0–15	5
QPlogKp	−8.0–−1.0	−3.625	#NandO	2–15	6
CNS	−2 (inactive), +2 (active)	−2	accptHB	2.0–20.0	6.25
QPlogBB	−3.0–1.2	−1.813	dipole	1.0–12.5	8.096
#amidine	0	0	IP(eV)	7.9–10.5	9.208
#amine	0–1	0	QPlogPw	4.0–45.0	13.562
#amide	0–1	0	QPlogPC16	4.0–18.0	13.867
#rtvFG	0–2	0	#ringatoms	N/A	16
#stars	0–5	0	#in56	N/A	16
SAfluorine	0.0–100.0	0	QPlogPoct	8.0–35.0	21.294
SAamideO	0.0–35.0	0	#nonHatm	N/A	26
RuleOfThree	maximum is 3	0	QPPCaco	<25 poor, >500 great	26.39
RuleOfFive	maximum is 4	0	QPPMDCK	<25 poor, >500 great	30.436
#in34	N/A	0	FOSA	0.0–750.0	35.632
#noncon	N/A	0	QPpolrz	13.0–70.0	39.832
Jm	N/A	0.000183	WPSA	0.0–175.0	71.346
ACxDN^.5/SA	0.0–0.05	0.0139316	PercentHumanOralAbsorption	>80% is high, <25% is poor	72.416
dip^2/V	0.0–0.13	0.057289	PSA	7.0–200.0	123.474
QPlogKhsa	−1.5–1.5	0.082	FISA	7.0–330.0	208.59
glob	0.75–0.95	0.786111	PISA	0.0–450.0	357.365
#acid	0–1	1	mol MW	130.0–725.0	370.791
EA(eV)	−0.9–1.7	1.037	SASA	300.0–1000.0	672.933
#metab	1–8	2	volume	500.0–2000.0	1144.025
HumanOralAbsorption	N/A	2	Compound	N/A	Small

## Data Availability

The manuscript provides all the data in figures, tables and Supplementary Materials.

## Supplementary Materials

The following supporting information can be downloaded at: https://www.mdpi.com/article/10.3390/ph18060772/s1, Figure S1. Optimisation results with various energies computed during the DFT computations for the Rebamipide compound.
