# Supplementary material for: Unveiling the Polypharmacological Potency of FDA-Approved Rebamipide for Alzheimer’s Disease
Source: Pharmaceuticals (Basel). 2025 May 22;18(6):772. doi: 10.3390/ph18060772 (PMC12196254; doi:10.3390/ph18060772)
Supplement: Supplementary file 1 [file pharmaceuticals-18-00772-s001.zip › pharmaceuticals-3560907-supplementary.pdf]

Optimisation with Jaguar:

The results of the Density Functional Theory (DFT) computations performed using the Jaguar module in Schrödinger Maestro provide a comprehensive analysis of the molecular properties of the compound. The compound Rebamipide, with the molecular formula $C_{19}H_{15}ClN_{2}O_{4}$, has a molecular weight of 370.79 g/mol and an exact mass of 370.0720347. It comprises 41 atoms, 4 hydrogen bond acceptors and 3 hydrogen bond donors, with no formal charge. The compound exhibits a bioavailability score 1, adheres to the Ghose filter, and complies with Lipinski's Rule of Five, indicating favourable drug-like properties. It contains 3 rings and 5 rotatable bonds, with a polar surface area of 95.5 Å^2, suggesting moderate polarity. The calculated ALOGPS_LOGP value of 2.32 and ALOGPS_LOGS value of -4.56 indicate moderate lipophilicity and low solubility (1.03e-02 g/l), respectively. The compound's logP (JCHEM_LOGP) is 2.76, further confirming its lipophilic nature. The electronic properties of the compound were analysed in detail. The highest occupied molecular orbital (HOMO) energy is -0.224 Hartree, while the lowest unoccupied molecular orbital (LUMO) energy is -0.059 Hartree, indicating a moderate energy gap. The average polarisability of the molecule is 36.82 Å^3, reflecting its ability to undergo electronic distortion. The compound's pKa values reveal its acidic and basic characteristics, with the strongest acidic pKa at 3.52 and the strongest basic pKa at -1.10. The average local ionisation energy (ALIE) analysis shows a maximum value of 328.61 Kcal/mol, a mean of 257.29 Kcal/mol, and a minimum of 201.36 Kcal/mol, indicating regions of varying electron density and reactivity. The electrostatic potential (ESP) analysis reveals a mean ESP of -61.85 Kcal/mol, with a maximum of 13.70 Kcal/mol and a minimum of -164.86 Kcal/mol, highlighting the charge distribution across the molecule. The DFT calculations used the B3LYP-D3 functional with the 6-31G** basis set, incorporating Grimme's D3 dispersion correction. The gas-phase energy of the compound is -1603.250542 Hartree, while the solvation energy in water is -64.18 Kcal/mol, resulting in a final solution-phase energy of -1603.352818 Hartree. The solvation energy indicates favourable interactions with the aqueous environment. The spin multiplicity of the compound is 1, confirming its singlet ground state. The geometry convergence category of 4 indicates a well-optimised structure. The compound's natural transition orbitals (NTOs) and excited states were analysed using time-dependent DFT (TDDFT), providing insights into its electronic transitions. The Polarisable Continuum Model (PCM) was employed to simulate solvation effects, with water as the solvent. The Quantum Mechanics (QM) Convergence Monitor ensured accurate interpretation of the results, confirming the reliability of the computed properties (Supplementary Figure S1).

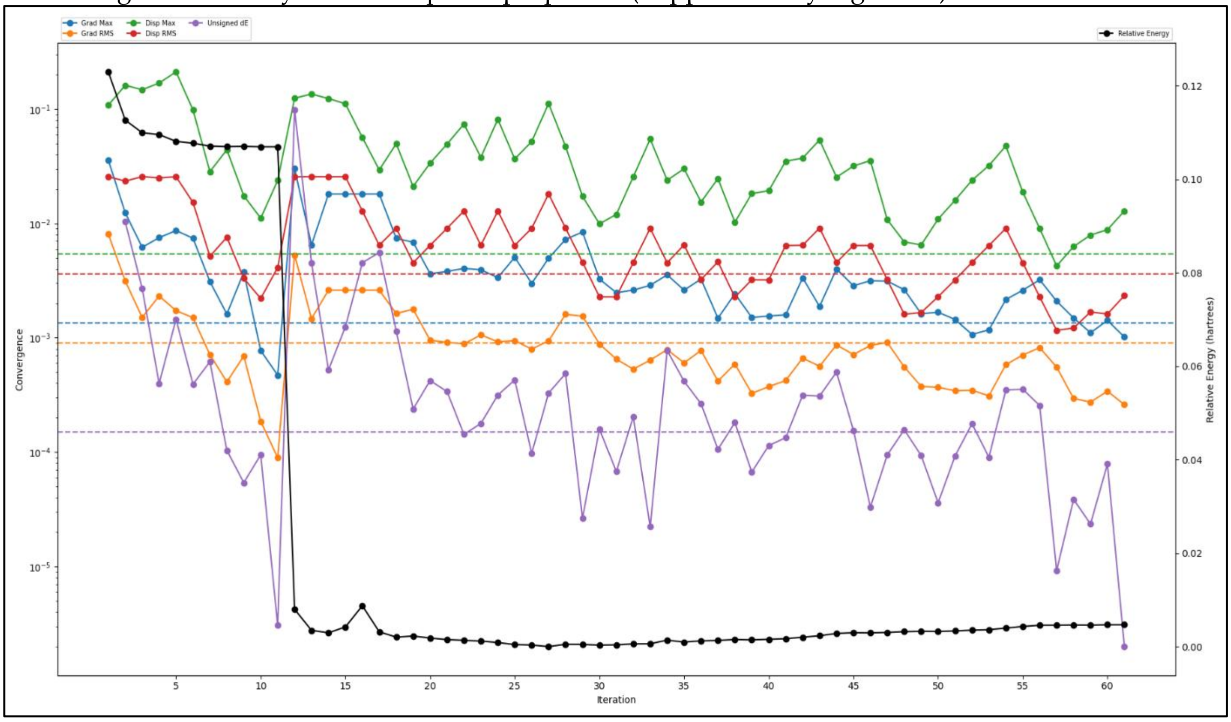

Supplementary Figure S1. Optimisation results with various energies computed during the DFT computations for the Rebamipide compound.
